# Familial Hypercholesterolemia: new therapeutic approaches

**DOI:** 10.1186/1824-7288-41-S2-A6

**Published:** 2015-09-30

**Authors:** Andrea Bartuli, Marina Macchiaiolo, Ippolita Rana, Paola Sabrina Buonuomo

**Affiliations:** 1 Rare Diseases and Medical Genetics, Department of Pediatrics, Bambino Gesù Children's Hospital IRCCS, Rome, Italy

## 

Familial Hypercholesterolemia (FH) is caused by a mutation in the gene that encodes the low-density lipoprotein (LDL) receptor, resulting in very high levels of circulating LDL-cholesterol and endothelial damage during time. Apoprotein B, activated by LDL receptor, and PCSK9 (proprotein convertase subtilisin / kexin type 9 serine Protease), that impairs the clearance of LDL receptors, are important additional mechanisms that lead to increase LDL-cholesterol.

The prevalence of Homozigous form (HoFH) is estimated to be 1 per 1 million individuals and, if untreated, proximal coronary disease or aortic valve disease frequently occurs during childhood and most affected patients suffer from fatal coronary disease before the age of 30. Heterozygous condition (HeFH) occurs in about 1/380-500 individuals. The levels of LDL-cholesterol in this form may greatly vary but the disease is always asymptomatic until adult age when there is a high risk of early cardiovascular disease.

Lifestyle intervention and maximal statin therapy are the mainstays of treatment. In patients that not achieve LDL-C targets, primarly HoFH patients, adjunctive lipoprotein apheresis is recommended where available.

Biological agents represents to date a new therapeutic approach for this not so rare condition.

The PCSK9 pathway is one of the best examples of how genetics has led to identification of a new target for cholesterol management.

Different **PCSK9 inhibitors** (alirocumab, evolocumab) are now available [1].

Mipomersen is an **antisense oligonucleotide (ASO)** that targets apoliporotein B-100 mRNA and disrupts its function: it is distributed mainly to the liver where it silences apoB mRNA, thereby reducing hepatic apoB-100 and giving rise to reductions in plasma total cholesterol, LDL-cholesterol, and apoB concentrations [2].

Lomitapide is a **microsomal triglyceride transfer protein (MTP) inhibitor**. MTP resides in the lumen of the endoplasmic reticulum, thereby preventing the assembly of apo B–containing lipoproteins in enterocytes and hepatocytes. This inhibition leads to a reduction in the synthesis of chylomicrons and very low–density lipoprotein, resulting in a reduction in plasma LDL levels [3].

## References

[B1] Huynh K Dyslipidaemia. Assessing the efficacy and safety of evolocumab and alirocumab Nat Rev Cardiol 2015 12 261 25824512 10.1038/nrcardio.2015.51

[B2] Gouni-Berthold I Berthold HK Mipomersen and lomitapide: Two new drugs for the treatment of homozygous familial hypercholesterolemia Atheroscler Suppl 2015 18 28 34 25936301 10.1016/j.atherosclerosissup.2015.02.005

[B3] Raper A Kolansky DM Sachais BS Long-term clinical results of microsomal triglyceride transfer protein inhibitor use in a patient with homozygous familial hypercholesterolemia J Clin Lipidol 2015 9 107 12 10.1016/j.jacl.2014.08.005 25670368 PMC4563802

